# Four New Species of *Harringtonia*: Unravelling the Laurel Wilt Fungal Genus

**DOI:** 10.3390/jof8060613

**Published:** 2022-06-08

**Authors:** João P. M. Araújo, You Li, Tuan A. Duong, Matthew E. Smith, Sawyer Adams, Jiri Hulcr

**Affiliations:** 1 Institute of Systematic Botany, The New York Botanical Garden, New York, NY 10458, USA; 2 School of Forest, Fisheries and Geomatics Sciences, University of Florida, Gainesville, FL 32611, USA; yourreason@hotmail.com (Y.L.); forestentomology@ifas.ufl.edu (S.A.); 3 Department of Biochemistry, Genetics & Microbiology, Forestry and Agricultural Research Institute (FABI), University of Pretoria, Pretoria 0002, South Africa; tuan.duong@up.ac.za; 4 Department of Plant Pathology, University of Florida, Gainesville, FL 32611, USA; trufflesmith@ufl.edu

**Keywords:** Ophiostomatales, Ophiostomataceae, ambrosia beetles, symbiosis, *Raffaelea*

## Abstract

Symbiosis between beetles and fungi arose multiple times during the evolution of both organisms. Some of the most biologically diverse and economically important are mutualisms in which the beetles cultivate and feed on fungi. Among these are bark beetles and *Harringtonia*, a fungal genus that produces *Raffaelea*-like asexual morph and hosts the causal agent of laurel wilt, *H. lauricola* (formerly *Raffaelea lauricola*). In this study, we propose four new species of *Harringtonia* associated with beetles from Belize and Florida (USA). We hope to contribute towards a more robust and inclusive phylogenetic framework for future studies on these beetle-fungi relationships and their potential impact in crops and forests worldwide.

## 1. Introduction

One of the hallmarks of fungi is their propensity to form intimate associations with other groups of organisms, including the most speciose group of animals on Earth, the insects [1,2,3]. Arthropods were among the first animals to colonize and exploit terrestrial ecosystems, an estimated 480 million years ago (mya) [4]. However, the most speciose group, the beetles, only appeared in the Permian around 280 mya [4]. Since the origin of the beetles, they have diversified into a variety of groups exhibiting a myriad of ecologies. One of the insect groups that has evolved close associations with fungi are the weevils (Coleoptera: Curculionidae), especially the subfamilies Platypodinae (pinhole borers) and Scolytinae (ambrosia and bark beetles) [5]. The obligate mycophagous lineages evolved repeatedly at least 12 times within these groups [6], the first at around 60 mya, and diversified into more than 4000 species. The obligate mycophagous beetles (also known as ambrosia beetles) share the same ecology of inhabiting wood, constructing galleries, introducing fungal inoculum into the tree hosts, and obligately feeding on the fungus as larvae [5]. Most of the fungal symbionts nutritionally associated with these beetles have been described in the genera *Raffaelea* Arx & Hennebert (Ophiostomatales) and *Ambrosiella* Brader ex Arx & Hennebert (Microascales) [7,8]. However, the ambrosial habit evolved in other fungal groups as well, such as *Flavodon* spp. (Basidiomycota: Polyporales, [9]), the *Fusarium ambrosium* clade (Hypocreales; [10]), and *Geosmithia* (Hypocreales, [11]). These intimate ambrosial symbioses between insects and fungi have resulted in the evolution of morphological adaptations in both groups, for example the mycangia (fungus pockets) in beetles and the ambrosial cells in fungi [12].

In the ambrosial symbiosis both beetles and fungi benefit from the obligate partnership. The fungus benefits from the insect because it is transmitted and inoculated into the tree host and the tunnels created by the beetle in the plant tissue allow the fungi to rapid penetrate into the substrate. The beetle mycangium, a pouch-like structure that evolved independently multiple times in different beetle lineages, serves to store and transport fungal propagules from one tree host to the next. These structures also play an important role by providing protection for the fungal propagules against desiccation during flight and hibernation of adult beetles [12,13]. The yeast-like fungal propagules multiply within the mycangia, producing pseudomycelium that serves as inoculum to colonize new tree hosts. On the other hand, the insect benefits by being exclusively mycophagous, thus deriving all its nutrition from the fungus [14,15].

Trees have evolved mechanisms to defend themselves against insects and fungi, primarily by producing specialized chemicals, resins and latexes. As a result, only a few of these fungus-carrying beetle lineages are able to colonize living, healthy trees in their natural habitat [16]. However, several of these beetles and fungi have become invasive species and caused great ecological and economic impacts [17] by colonizing naïve trees which have not evolved with these unusual pathogens, such as avocado [18].

Among these fungi carried by the beetles, *Harringtonia lauricola* (T.C. Harr., Fraedrich & Aghayeva) Z.W. de Beer & M. Procter (formerly *Raffaelea lauricola*) stands out as an important systemically pathogenic ambrosia fungus [7,18]. It has caused substantial mortality of non-native, hyper-sensitive host trees by moving through the vessels of the tree host and causing a vascular wilt disease in redbay (*Persea borbonica*), avocado (*Persea americana*) and other North American members of the Lauraceae family. Because the susceptible hosts belong to Lauraceae, the disease caused by this fungus is referred to as “laurel wilt” [18]. The pathogen is native to East Asia, where, vectored by the ambrosia beetle *Xyleborus glabratus*, it colonizes injured lauraceous hosts. After it invaded the Southeastern U.S. in the early 2000’s, it spread rapidly as far as Texas [19]. It remains unclear whether this fungus is unique in its virulence, of if the Asian ambrosial Funga includes other species with potential for similar devastating invasions.

The family Ophiostomataceae includes three closely related ambrosial genera which were all until recently included in the polyphyletic genus *Raffaelea* [20] The largest of these three genera is *Raffaelea* s. str., which includes dozens of ambrosia fungi associated with numerous unrelated groups of ambrosia beetles. *Dryadomyces* (Gebhardt) Z.W. de Beer & M. Procter (former the *R. sulphurea* complex) includes a handful of ambrosia symbionts of Scolytinae (mostly Xyleborini) and Platypodinae, such as *D. quercivorus* Kubono & Shin. Ito (implicated as a mortality factor in Japanese oak wilt) and *D. quercus-mongolicae* K.H. Kim, Y.J. Choi & H.D. Shin (Korean oak wilt). The third clade, *Harringtonia* (former *R. lauricola* complex), currently includes only three species: *H. aguacate* D.R. Simmons, Dreaden & Ploetz, *H. brunnea* (L.R. Batra) T.C. Harr. and the infamous *H. lauricola* [21].

In this study, we present four new species belonging to the genus *Harringtonia*. All these species were isolated from mycangia of four species of beetles from Belize and the USA. Given the ecological and economical importance of some *Harringtonia* species, particularly *H. lauricola*, it is important that we recognize and characterize other species belonging to this group since they may represent important potential threats to forests and agriculture. For that reason, we not only described these new species, but also conducted pathogenicity tests on redbay, a tree species from the Southeastern USA that is highly susceptible to *H. lauricola*. The combination of taxonomy, a resolved phylogenetic framework, and host interaction data will allow for a better understanding of the evolutionary biology of the ambrosial symbioses.

## 2. Material and Methods

### 2.1. Fungus Isolation

We isolated *Harringtonia* associates from *Dryocoetoides capucinus* (Curculionidae: Scolytinae: Xyleborini), *Euplatypus longius* and *Megaplatypus godmani* in Belize and *E. parallelus* in the USA (Florida) (Curculionidae: Platypodinae) (Table 1). Whole beetles were surface-washed by vortexing for 1 min in 1 mL of sterile distilled water with 1 small drop of Tween detergent. Sampling focused on recovering fungi from the body parts of adult ambrosia beetles that include their mycangia: pronota of adult platypodines and the head of *Dryocoetes* beetles were removed and crushed in a 500 µL of sterile phosphate buffer saline and vortexed for 30 s. The resulting solutions were diluted to 1:10, 1:100 and 1:1000 concentrations, and each dilution was used to inoculate potato dextrose agar (PDA; Becton, Dickinson and Company, Sparks, MD, USA) plates. Fungi were allowed to grow at 25 °C for 5–10 d. Representative isolates of different fungal morphotypes were placed onto new 2% PDA plates to obtain pure cultures and these were retained for molecular identification. Axenic cultures of the fungi are deposited in the culture collection (CMW) of the Forestry and Agricultural Biotechnology Institute (FABI), University of Pretoria, South Africa and in the culture collection (CBS) of Westerdijk Fungal Biodiversity Institute, Utrecht, The Netherlands.

### 2.2. Morphological Studies

For morphological characterization, we collected small samples of each isolate in 3–5 parts across the plate, i.e., edge, intermediate portion and center. The fungal samples were mounted in 4% lactic acid and observed under optical microscope equipped with DIC (Nomarski) capabilities (Zeiss Axioscope 5). Measurement of taxonomically informative structures, e.g., conidiogenous cells, conidia, chlamydospore, etc., was performed using the Zen software (Zeiss, Jena, Germany). The semi-permanent slides were sealed with nail polish by direct applications of at least 3 layers around the cover slip edges and stored in a slide box for further observations.

### 2.3. Taxa Sampling and Sources

To investigate the relationship of *Harringtonia* species with other genera within Ophiostomatales, we constructed a phylogenetic tree based on ITS, LSU and ß-tubulin sequences using DNA data from [22,23], resources available in GenBank and our new isolates. The dataset consisted of 73 sequences from ophiostomatalean species in several genera (*Leptographium* (19), *Grosmannia* (4), *Esteya* (3), *Dryadomyces* (8), *Raffaelea s.s.* (23), *Harringtonia* (14)) as well as the investigated isolates [9], and two outgroup taxa (*Sporothrix eucalypterigena* and *Ophiostoma piliferum*) (Table 2).

### 2.4. DNA Extraction, PCR and Sequencing

Extraction of genomic DNA was performed by scraping 5–10 mg fungal mycelium from pure cultures and adding it to 20 μL extraction solution from the Extract-N-Amp Plant PCR kit (Sigma-Aldrich, St. Louis, MO, USA). Samples were then incubated at 96 °C for 30 min. After the incubation period, 20 μL of 3% bovine serum albumin solution was added, and the mixture was vortexed and centrifuged at 6000 rpm for 20 s. The supernatant was used as template for PCR amplification.

Three gene regions including the nuclear large subunit (28S), ITS, and β-tubulin (βT) were amplified and sequenced. Primer combinations used for amplifications were LR0R and LR5 [24,25] for 28S; ITS1 or ITS1f and ITS4 for ITS [26,27] and T10 or Bt2a and Bt2b [28,29] for βT. The PCR conditions for ITS and βT were the same as those used by [30], i.e., an initial denaturation step at 95 C for 5 min, followed by 35 cycles of 95 °C for 30 s, 53 °C annealing for 30 s, 72 °C extension for 60 s and a final extension step at 72 °C for 8 min. The sequencing was performed with both forward and reverse primers as used in PCR. For the 28S regions we used the similar PCR conditions except the 55 °C annealing for 45 s following Li et al. [9] Amplified products were visualized and purified as described by [21], and these were submitted to GENEWIZ (South Plainfield, NJ, USA) for sequencing. Sequence chromatograms were inspected for quality and assembled in Geneious v. 9.1.5 (www.geneious.com) (accessed on 15 March 2022).

### 2.5. Phylogenetic Analyses

Individual alignments were performed for each locus with MAFFT v. 7.450 [31]. The alignment for each individual locus was improved manually by trimming the ends. The sequences were then annotated and concatenated into a single combined dataset using Geneious v. 11.1.5 [32]. Ambiguously aligned regions were excluded from phylogenetic analysis and gaps were treated as missing data. The final alignment length was 3252 bp: 1332 bp for ITS, 893 bp for LSU and 1027 bp for β-tubulin. Maximum likelihood (ML) analyses were performed with RAxML v. 8.2.4 [33] on the concatenated dataset containing all three loci. The dataset consisted of five data partitions, including one each for LSU and ß-tubulin and three for ITS (ITS1, 5.8S and ITS2). The GTRGAMMA model of nucleotide substitution was employed and 1000 bootstrap (BP) replicates were conducted.

### 2.6. Pathogenicity Test

To test whether the new *Harringtonia* spp. have similar level of pathogenicity as *H. lauricola*, we tested their effect on redbay (*Persea borbonia*). Seedling were provided by Half Moon Growers (Micanopy, FL, USA). The initial height of the containerized tree (13 L container) was about 1.2–1.3 m with a trunk diameter of 1.0–1.7 cm at 5.0 cm above soil level. All trees were stored and tested in a quarantine greenhouse, a biosafety level 2 (BSL2) facility at the Division of Plant Industry (DPI), Department of Agriculture and Consumer Services in Gainesville, FL, USA under the USDA/APHIS permit No. P526P-16-02872. Each tree was grown in a 3-gallon pot and was examined for 2 weeks before inoculation to ensure the absence of any disease symptom caused by plant pathogens or insects. The seedlings were maintained under natural light conditions, watered daily, and kept under a day–night temperature regime averaging at 27 °C. No additional treatments (e.g., fertilization or pesticide) were applied.

Tree inoculations were made to simulate an ambrosia beetle boring with fungal spores by drilling at a downward angle (approx. 45 degrees) into the xylem of each seedling using a 2.38 mm drill bit. Holes were made within the basal 5 cm of the stem and were up to 10-mm deep. Spore suspensions were pipetted into the xylem in 50 µL of aliquots. To prepare the inoculum, fungal isolates were cultured onto PDA. The number of spores in suspensions was normalized to the maximum number of colony-forming units (CFU) obtained for each beetle species. Clean water was used as negative control and *Raffaelea lauricola* was used as positive control. Three tree replicates were inoculated with each respective isolate. After inoculation, wounds were wrapped with parafilm immediately to avoid cross contamination.

Seedlings were monitored weekly, recording all external signs and symptoms (including sap bleeding, canker development, and mortality). To quantify the extent of fungal infection and the host response, trees were destructively sampled 10 weeks after inoculation. Bark was peeled near the hole of inoculation using a carpet knife. Then, trees were cut longitudinally, through the point of inoculation, to uncover the sapwood staining area. The discolored xylem (stain length) was measured using a caliper and a transparent soft ruler. Finally, to fulfill Koch’s postulates, isolations were made from the discolored wood surrounding the inoculation site, rinsing in sterile water, blotting dry, plating onto PDA plates, and incubating at 25 °C. Cultures which resemble the morphology of the inoculated fungi were sub-cultured onto PDA plates, and then identified using morphology or PCR and sequencing.

### 2.7. Data Analysis

Pathogenicity test data were processed by using Microsoft Office Excel 365 ProPlus and differences in the size of discolored lesions relative to the positive control was tested using analysis of variance (ANOVA) and T-test by GraphPad Prism 7.

## 3. Results

### 3.1. Phylogenetic Analyses

**Figure 1:**
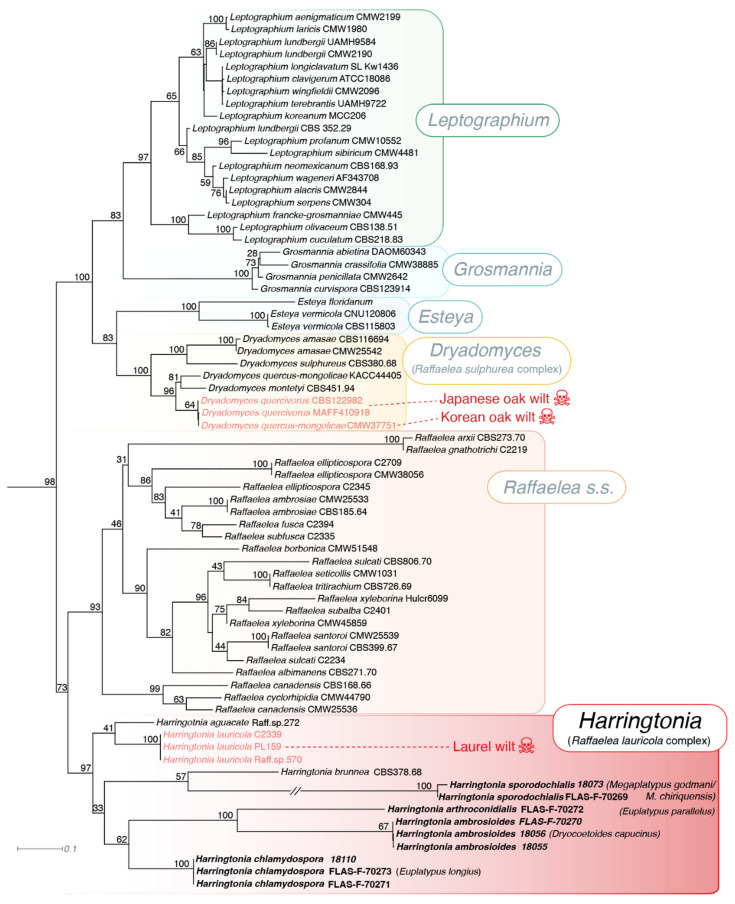
Maximum likelihood tree obtained from RAxML analysis of the concatenated dataset composed by LSU, ITS and ß-Tubulin of ophiostomatalean species. Our dataset was composed of 73 isolates and final concatenated alignment consisting of 3253 bp. All bootstrap values are shown. The new species of *Harringtonia* proposed are in bold. Numbers near genus and species epithets refer to isolate numbers and vector beetle species are indicated to the right of the four new species of *Harringtonia*. Note: *Megaplatypus godmani*/*chiriquensis* refers to an ambiguous beetle ID, not two different beetle species being associated with *H. sporodochialis*.

Our phylogenetic analyses based on ITS, LSU and ß-tubulin corroborated previous studies that have resolved relationships within and between ophiostomatalean genera [20,22,23]. We recovered three monophyletic clades containing species traditionally considered within the genus *Raffaelea* (Figure 1). The first, *Dryadomyces* (BP = 83), is placed within a larger group composed of other non-ambrosial fungi in the genera *Esteya*, *Grosmannia* and *Leptographium*. The genera *Raffaelea s.s*. (BP = 93) and *Harringtonia* (BP = 97) each formed their own strongly supported monophyletic clade and together these two lineages formed a larger, marginally well supported monophyletic group (BP = 73). All four of the new proposed species were resolved within the genus *Harringtonia*. Descriptions of these four new taxa are provided below.

### 3.2. Taxonomy

**Figure 2:**
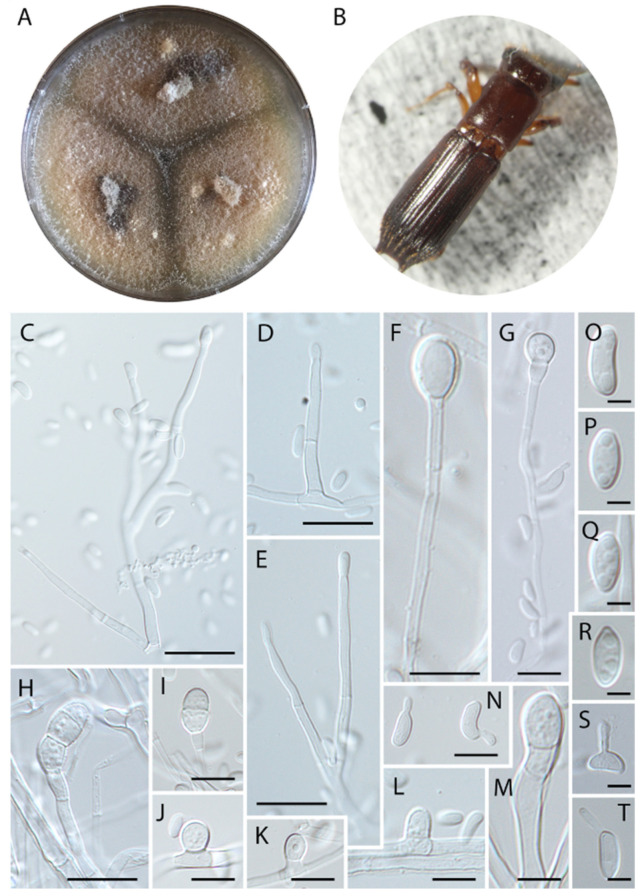
*Harringtonia chlamydospora* in pure culture on PDA. (**A**) Colony growth after 3 weeks at 25 °C. (**B**) Beetle vector, *Euplatypus longius*. (**C**) Terminal phialide. (**D**) Lateral phialide. (**E**) Terminal phialide. (**F**,**G**) Early stages of terminal chlamydospores. (**H**,**I**) Fully developed chlamydospore. (**J**–**L**) Micronematous conidiogenous cells formed laterally on the vegetative hypae. (**M**) Chlamydospore. (**N**) Germinating conidia. (**O**–**R**) Conidia. (**S**,**T**) Conidia exhibiting germ tube. Scale bars: (**C**–**E**) = 10 µm, (**F**,**G**) = 20 µm, (**H**) = 20 µm, (**I**) = 10 µm, (**J**–**L**) = 5 µm, (**M**) = 10 µm, (**N**) = 5 µm, (**O**–**T**) = 2 µm.

***Harringtonia chlamydospora*** Araújo, Y. Li & J. Hulcr, sp. nov.–Mycobank (MB844121) (Figure 2).

***Etymology.*** The epithet “chlamydospora” refers to the uncommon terminal, septate chlamydospores formed in this species.

***Typus*.** Belize, Cayo prov., Las Cuevas research station, 16.7331 N, 88.9862 W, from gallery of *Euplatypus longius* mycangium, 29 January 2019, collected by YL and JH (holotype: FLAS-F-70271; isotype: FLAS-F-70273).

***Diagnosis*.** Fungus associated with *Euplatypus longius* mycangium, inhabiting *Zanthoxylum* sp. (Rutaceae), exhibiting typical septate chlamydospores of 8–21 × 5–7 µm. Fungus producing typical terminal chlamydospore in culture.

***Description*.** Colonies initially cream, turning brown with age; reverse light orange on PDA. Fungi rapidly occupying the entire plate. Sexual morph was not observed. Asexual morph was composed of cylindrical, hyaline, regularly septate hyphae, ranging from 2–4 µm thick. Three types of conidiation were observed. The first type is sessile, micronematous, laterally forming globose to hemispherical conidia, sometimes leaving a scar after conidium release, formed solitarily and directly on the vegetative hyphae, 5–6.5 × 3.5–5 µm (Figure 2J–L). The second type are terminally formed chlamydospores, hyaline, cylindrical, with 0–2 septa, developing a slightly verrucose wall with age (Figure 2H), 8–21 × 5–7 µm (Figure 2F–I,M). The third type formed on hyaline phialides, produced terminally or laterally on the main hypha, erect, occasionally irregular, cylindrical, sometimes tapering slightly towards the apex, (15–) 21 (–30) × 2.5 µm (Figure 2C–E). Conidia hyaline, produced singly, aseptate, globose to elongate, sometimes curved, smooth-walled, commonly exhibiting germ tubes, (3.5–) 6 (–7) × 2.5–3 (–4) µm (Figure 2S,T).

***Vector*.** Thus far only known from *Euplatypus longius* but the actual range of vectors is not known.

***Host.*** The only recorded host is a dead *Zanthoxylum* sp. (Rutaceae), but the host range is likely broader.

***Distribution.*** Thus far only known from Belize, the full distribution is unknown.

***Additional specimens examined.*** Belize, Cayo prov., Las Cuevas research station, in *Euplatypus longius* mycangium, 29 January 2019, collected by YL and JH (FLAS-F-70273).

***Note.****Harringtonia chlamydospora* differs from its closely related *H. arthroconidialis* and *H. ambrosioides* by the presence of multiple types of conidiation, and the typical terminal chlamydospore.

**Figure 3:**
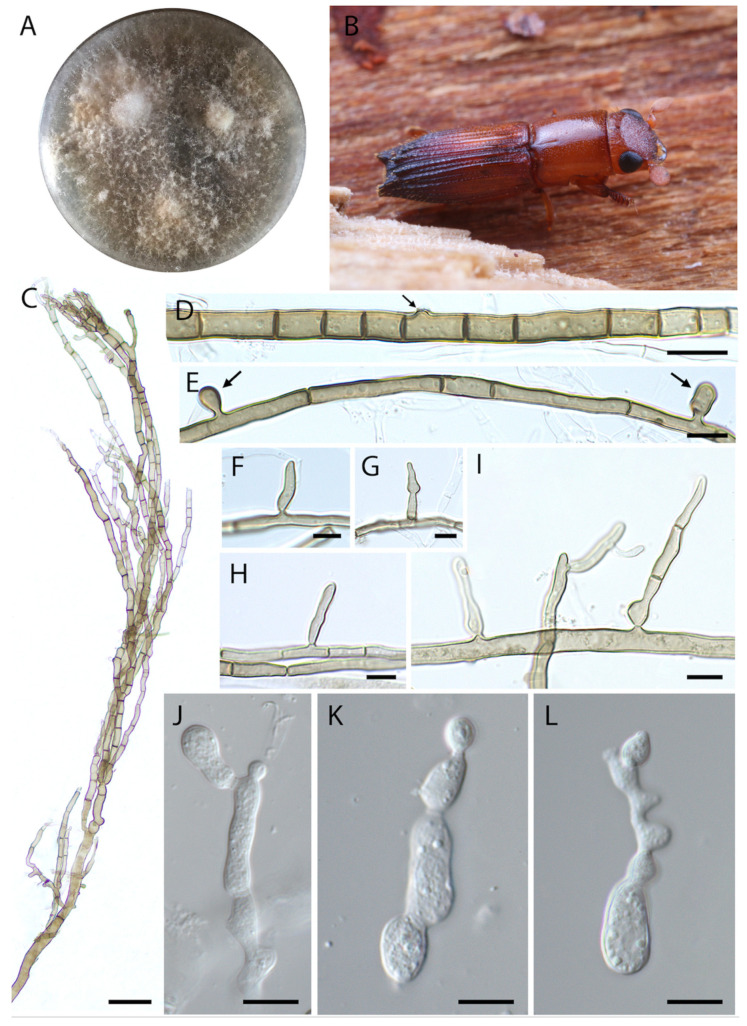
*Harringtonia arthroconidialis* in pure culture on PDA. (**A**) Colony growth after 3 weeks at 25 °C. (**B**) Beetle vector, *Euplatypus parallelus*. (**C**) Branched olivaceous hyphae. (**D**) Formation of arthroconidia, arrow indicates site of conidium attachment. (**E**) Micronematous conidiation (early stage). (**F**–**H**) Sessile conidia. (**I**) Conidia that remained attached forming 1–3 celled propagules. (**J**–**L**) Yeast-like cells budding. Scale bars: (**C**) = 20 µm, (**D**,**E**) = 10 µm, (**F**–**I**) = 5 µm, (**F**–**I**) = 10 µm.

***Harringtonia arthroconidialis*** Araújo, Y. Li & J. Hulcr, sp. nov.–Mycobank (MB844122), Figure 3.

***Etymology***. Epithet refers to the arthroconidia produced by this species.

***Typus***. Miami, FL, USA, Tropical Research and Education Center, Miami-Dade, 25.5077 N, 80.5035 W, in a male *Euplatypus parallelus* head, 10 June 2018, collected by YL and JH (holotype: FLAS-F-70272). Beetle vectors captured in light traps.

***Diagnosis***. Fungus associated with beetles, exhibiting olivaceous vegetative hyphae, arthroconidia 10–11 × 5.5–6.5 µm and yeast-like cells.

***Description***. Colonies were initially white, turning cream to olivaceous with age, aerial mycelium loose; reverse cream, darkening towards the inoculation point. Sexual morph not observed. Asexual morph composed of cylindrical hyphae, irregular, 2–5 µm thick, hyaline to olivaceous-brown, thick-walled, branched, irregularly septate. Two types of conidia observed. Conidiophores micronematous. Conidia formed laterally on the vegetative hyphae, sessile, cylindrical, elongated, solitary, irregular, sometimes swollen and tapering towards the apex (Figure 3G), (10–) 13–16 × 2.5–4 µm (Figure 3E–I). Conidia remaining temporarily attached to the main hyphae at maturity, forming three-cell propagules averaging 35 × 4 µm (Figure 3I). Arthroconidia formed by the septation of olivaceous vegetative hyphae, up to 20 units of (6.5–) 10–11 (18.5–) × 5.5–6.5 µm (Figure 3D). Yeast-like cells were commonly present, usually ovoid to cylindrical with round ends, thin walled, 10–30 × 5–15 µm, producing daughter cells that may become detached or remain attached (Figure 3J–L).

***Vector.*** Thus far known only from *Euplatypus parallelus* but the actual range of vectors is not known.

***Host.*** Unknown (beetle vector collected in trap).

***Distribution***. Miami, FL, USA.

***Note.****Harringtonia arthroconidialis* differs from its sister species, *H. ambrosioides*, by the formation of arthroconidia, and sessile propagules. It may also differ by association with *E. parallelus*.

**Figure 4:**
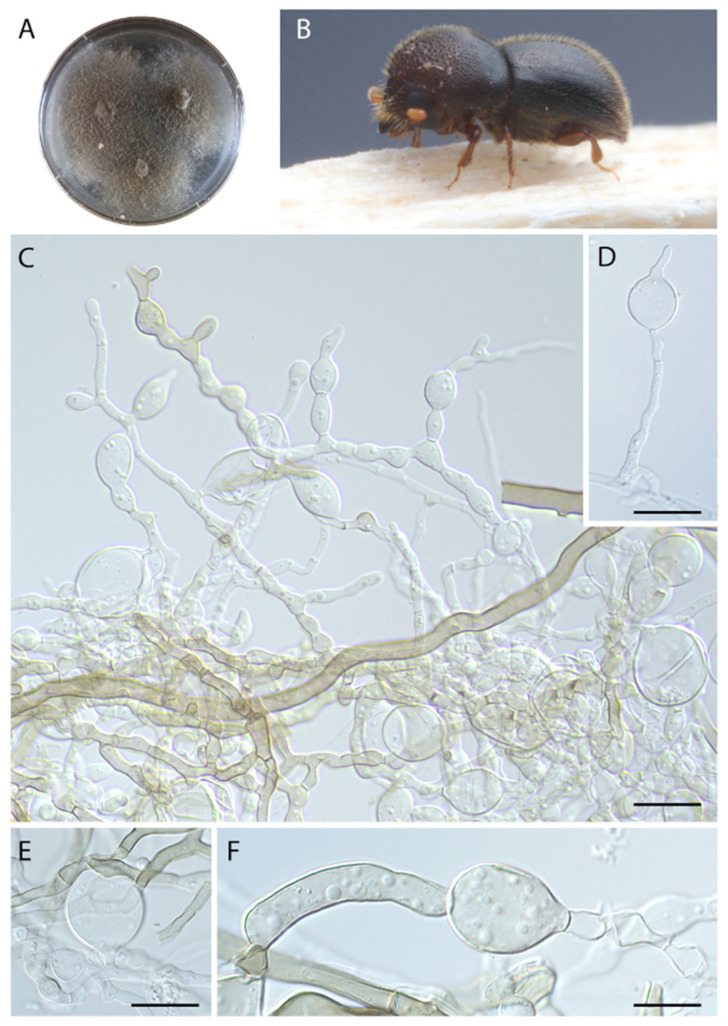
*Harringtonia ambrosioides* in pure culture on PDA. (**A**) Colony growth after 3 weeks at 25 °C. (**B**) Beetle vector, *Dryocoetoides capucinus*. (**C**) Aspect of vegetative hyphae bearing multiple ambrosial cells (vesicles). (**D**) Close-up of a terminal vesicle. (**E**) Vesicle produced laterally on the main hyphae. (**F**) Enlarged hyphae. Scale bars: (**C**) = 15 µm, (**D**) = 10 µm, (**E**) = 20 µm, (**F**) = 10 µm.

***Harringtonia ambrosioides*** Araújo, Y. Li & J. Hulcr, sp. Nov.–Mycobank (MB844123), Figure 4.

***Etymology.*** Name refers to the predominance of ambrosial cells, which are enlarged vesicles that serve as nutritional source for ambrosia beetle vectors.

***Typus*.** Belize, Cayo prov., Las Cuevas research station, 16.7331 N, 88.9862 W, in *Dryocoetoides capucinus* head, 31 January 2019, You Li (holotype: FLAS-F-70270).

***Diagnosis*.** Fungus associated with beetles, exhibiting abundant vesicles averaging 20 × 15 µm.

***Description*.** Colonies initially cream, turning olivaceous-brown with age. Sexual morph not observed. Asexual morph composed of sterile hyphae, hyaline to light olivaceous, regularly septate, composed by cylindrical hyphae that often turn into (14–) 20 (–28) × (9–) 15 (–25) µm vesicles (ambrosial cells). No conidiogenous cells observed.

**Vector.***Dryocoetoides capucinus*.

***Host.*** Plant host unknown (beetle collected in trap).

***Distribution*.** Only collected in Belize, the full distribution is unknown.

***Additional specimens examined***. Belize, Cayo prov., Las Cuevas research station, in *Dryocoetoides capucinus* heads, 31 January 2019, You Li (18055, 18056).

***Note.****Harringtonia ambrosioides* differs from its sister species, *H. arthroconidialis*, by the production of hyaline to olivaceous vesicles that could, potentially, work as dispersion units. It may also differ by association with *D. capucinus*, which is unrelated to the other ambrosia beetles of the subfamily Platypodinae sampled in this study.

**Figure 5:**
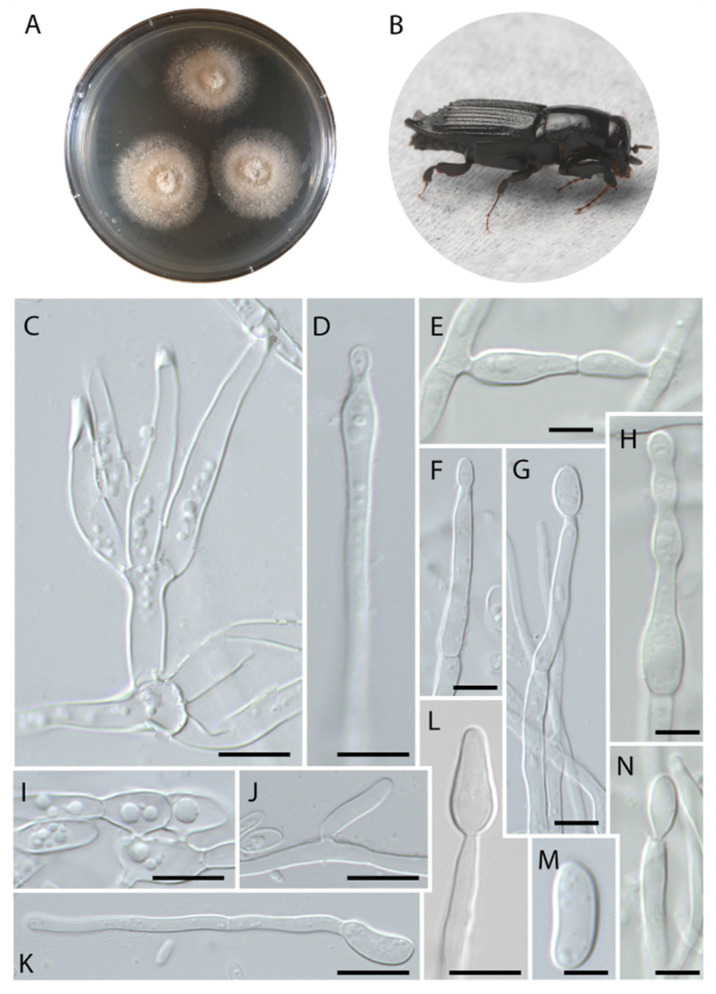
*Raffaelea sporodochialis* in pure culture on PDA. (**A**) Colony growth after 3 weeks at 25 °C. (**B**) Beetle vector, *Megaplatypus godmanii*. (**C**) Sporodochia composed of 4 phialides formed from a single basal cell. (**D**) Early developmental stage of aleuriospore. (**E**) Anastomosing hyphae. (**F**–**H**) Aleuriospores. (**I**) Anasmotomozing cells, presumably aiding transfer of nutrients throughout the hyphae. (**J**) Sessile conidia. (**K**) Germinating conidium. (**L**) Aleuriospore. (**M**) conidium. (**N**) Aleuriospore being produced at the hyphal tip. Scale bars: (**C**) = 10 µm, (**D**) = 5 µm, (**E**,**F**) = 3 µm, (**G**,**H**) = 5 µm, (**I**,**J**) = 10 µm, (**K**,**L**) = 5 µm, (**M**,**N**) = 3 µm.

***Harringtonia sporodochialis*** Araújo, Y. Li & J. Hulcr, sp. nov.–Mycobank (MB844124), Figure 5.

***Etymology.*** Epithet refers to the sporodochia produced by this fungus.

***Typus*.** Belize, Cayo prov., Las Cuevas research station, 16.7771 N, 89.0215 W, in mycangium of *Megaplatypus godmani* heads and pronotum; collected by YL and JH, 31 January 2019 (holotype: FLAS-F-70269).

***Diagnosis*.** Fungus associated with beetles, inhabiting the trunk of dead *Zanthoxylum* sp., exhibiting sporodochia in culture, composed by 3–4 phialides of 22 × 2.5–4 µm.

***Description*.** Colonies initially white, turning light cream with age; reverse sub-hyaline, light yellow to cream. Sexual morph not observed. Asexual morph composed of cylindrical, hyaline, regularly septate hyphae. Three types of conidiation were observed. The first type were sessile, micronematous, forming conidia laterally, directly on the vegetative hyphae, cylindrical, think-walled, usually truncate at the base and round at the apex, 12–18 × 2.5–3.5 µm (Figure 5J). The second type of conidia formed terminally on hyphal branches (aleuriospore), hyaline, smooth, globose to elongate, rarely curved, 8–15 × 4 –5.5 µm (Figure 5D,F–H,L,N). The third type of conidia formed within sporodochia composed by 3–4 erect phialides that emerge from a single basal cell, hyaline, slightly irregular, (16–) 22 (–28) × 2.5–4 µm. Conidia hyaline, globose to slightly elongated, sometimes curved, thin-walled, commonly found germinating, 5–8 × 3.5 µm (Figure 5K).

***Vector*.** Megaplatypus godmanii and M. chiriquensis.

***Host.*** Zanthoxylum sp.

***Distribution***. Belize.

***Additional species examined.*** Belize, Cayo prov., Las Cuevas research station, in mycangium of *Megaplatypus godmani*; collected by YL and JH, 31 January 2019 (18073 (to be assigned)).

**Note.***Harringtonia sporodochialis* differs from its sister species, *H. brunnea*, by the formation of sporodochia supported by a basal cell, the formation of sessile conidia and hyaline, thin-walled hyphae. The association with the beetle vector is also different: *M. godmani* with *H. sporodochialis* and *Monarthrum fasciatum*, *M. mali* and *M. scutellare* with *H. brunnea*. However, the actual host fidelity is unknown.

### 3.3. Pathogenicity Test

**Figure 6:**
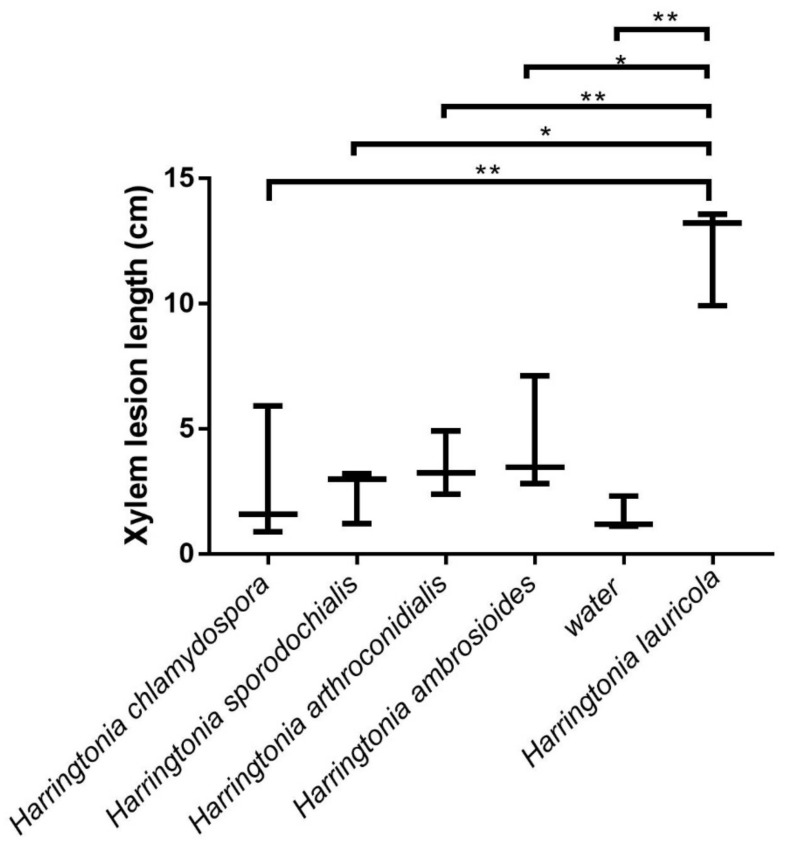
Lesion length response by red bay tree inoculated with different *Harringtonia* fungi and water. Bar of lesion length with the asterisks in each column indicated significant difference (* *p* < 0.05; ** *p* < 0.01) with Tukey HSD test.

*Harringtonia lauricola* is a very serious pathogen and, therefore, it is important to test whether its relatives also have the ability to cause serious disease in Lauraceae, or if this is a unique feature of *H. lauricola*. None of the four new *Harringtonia* species caused any symptoms of disease, external lesions, or death of red bay saplings when observed after 10 weeks post-inoculation. Positive control red bay saplings inoculated with *H. lauricola* all died within 10 weeks post-inoculation. Wilt was already observed on these positive control trees 15 days after inoculation. Although inoculation sites were discolored, none of the four new species were statistically different from the negative control water inoculations (Figure 6). In the inoculation treatments only *H. sporodochialis*, *H. chlamydospora* and *H. lauricola* were successfully re-isolated from the discolored wood near the inoculation site after 10 weeks.

## 4. Discussion

In the current study, we identified and characterized four distinct lineages of *Harringtonia* which are described here as the new species *H. ambrosioides*, *H. arthroconidialis*, *H. chlamydospora* and *H. sporodochialis* (Figure 1). *Harringtonia* species are clearly vectored by multiple lineages of ambrosia beetles (Scolytinae as well as Platypodinae), but our data are not sufficient to measure specificity in these associations. Further sampling may reveal some degree of promiscuity as noted in other species within this group [34].

*Harringtonia* fungi usually exhibit limited taxonomically informative characters [7]. However, *H. sporodochialis* and *H. chlamydospora* exhibit a broader variety of microscopic traits compared with other *Harringtonia* species because they form three different types of conidia when grown in pure culture. The micronematous conidial formation (similar to vegetative hyphae, Figure 2 J–L and Figure 5J) and phialides (Figure 2C–E and Figure 5F,G,L,N) occur in both species. Chlamydospores were observed only in *H. chlamydospora* (Figure 2F–H,M) and sporodochia were only observed in *H. sporodochialis* (Figure 5C), hence their respective epithets. *Harringtonia ambrosioides* exhibited peculiar enlarged vesicles. We hypothesize that these cells serve as the food source for beetle larvae and that they are functionally analogous to gongylidia produced by *Leucoagaricus* cultivated by leaf-cutting ants to also serve as a food source [35], but their functional as propagules (spores) should not be disregarded and this need to be addressed in future studies.

The genus *Harringtonia* is currently composed of seven species, all associated with wood boring ambrosia beetles, including the four new species described herein. These species form a monophyletic clade, which was until recently referred to as the *Raffaelea lauricola* group (Figure 1, [20]). Among *Harringtonia*, only *H. lauricola* is known to cause a serious plant disease, the “laurel wilt”. This systemic vascular disease affects New World trees in the family Lauraceae and has killed over a half-billion trees in just a decade [5,36]. The most widely accepted hypothesis is that these beetles were brought from Asia into USA through seaports in Savannah (Georgia), introduced in wood packing containers. The beetle (*X. glabratus*), and the fungus (*H. lauricola*) within its mycangia, became established and further expanded their range to neighboring states, and became serious invasive species across this range, particularly damaging in avocado orchards in south Florida [18]. Such a spread throughout the newly conquered environment was likely facilitated by the promiscuity displayed by the fungus, which is also capable of dispersal via other beetle species within the genus *Xyleborus* [21,33]. Thus, understanding the diversity of these fungi and how they interact with their beetle vectors is crucial for an effective diagnosis and development of strategies to control potential threats caused when exotic *Raffaelea*-carrying beetles are introduced to a certain habitat. The fact that we found three new species with a small amount of sampling in just one site in Belize hints that the diversity of these fungi is extremely understudied. Thus, it is urgent to broaden the sampling of these fungi in tropical forests in order to understand the species diversity and potential new threats in forests worldwide.

In the pathogenicity tests, none of the trees died and only minor symptoms were detected. This indicates that all new *Harringtonia* are not lethal to the trees tested. This result is similar to another *Harringtonia* fungus, *Raffaela aguacate*, which also does not produce the same pathogenic results as *H. lauricola* on swampbay tree *Persea palustris* [37]. However, even though all *Harringtonia* except *H. lauricola* appears to be not plant pathogens in the conditions investigated in the current study, the *Harringtonia*-ambrosia beetle interaction should not be considered harmless because polyphagous ambrosia beetles are often highly invasive. Some unknown or novel *Harringtonia* may appear to be non-pathogen in their native area but may become lethal to naive tree hosts or they may play a role in the mass accumulation of their beetle vectors when introduced in a new environment [35].

## Figures and Tables

**Table 1:** Morphological features, Vector, Host and Distribution information of *Harringtonia* species.

Species	Phialide (µm)	Conidia (µm)	Budding Conidia (µm)	Chlamydospore (µm)	Sporodochia	Vector	Host	Distribution
*Harringtonia aguacate*	13 × 2.7	7.2 × 2.6	Rare	–	–	*–*	*Persea americana*	FL (USA)
*Harringtonia lauricola*	13–60 × 2	4.5 × 1.5–2	+	–	–	*Xyleborus glabratus*	*Persea borbonia*	SC (USA)
*Harringtonia brunnea*	10–42 × 3–4.5	8–13 × 8–15	–	–	+	*Monarthrum fasciatum, M. mali, M. scutellare*	*Quercus, Acer*	MS (USA)
*Harringtonia sporodochialis*	16–28 × 2.5−4	5−8 × 3.5	+	–	+	*Megaplatypus godmanii*	?	Belize
*Harringtonia ambrosioides*	–	–	–	–	–	*Dryocoetoides capucinus*	?	Belize
*Harringtonia chlamidospora*	21 × 2.5	6 × 2.5–3	+	8–21 × 5–7 (Terminal)	–	*Euplatypus longius*	?	Belize
*Harringtonia arthroconidialis*	–	13–16 × 2.5–4 (arthroconidia)	+	–	–	*Euplatypus parallelus*	?	FL (USA)

**Table 2:** Species, Voucher and GenBank information of the species used in this study (Figure 1). The new species proposed in this study is highlighted in bold with types marked with *.

Species	Voucher	ITS	LSU	ß-Tubulin
*Dryadomyces amasae*	CBS116694	–	EU984295	EU977470
*Dryadomyces amasae*	CMW25542	–	MT629750	–
*Dryadomyces montetyi*	CBS451.94	–	EU984301	EU977475
*Dryadomyces quercivorus*	CBS122982	MT633072	MT629762	MT644090
*Dryadomyces quercivorus*	MAFF410918	–	AB496454	GQ225691
*Dryadomyces quercus-mongolicae*	KACC44405	MT633074	MT629763	–
*Dryadomyces quercus-mongolicae*	CMW37751	–	–	MT644091
*Dryadomyces sulphureus*	CBS380.68	MT633077	EU984292	EU977467
*Esteya floridanum*	18111	MT858361	LC363546	–
*Esteya vermicola*	CNU120806	–	EU627684	FJ490553
*Esteya vermicola*	CBS115803	–	EU668903	FJ490552
*Gorsmannia clavigerum*	ATCC18086	–	AY544613	AY263194
*Gorsmannia cucullata*	CBS 218.83	NR_145269	NG_064129	–
*Grosmannia abietina*	DAOM60343	DQ097852	–	AY263182
*Grosmannia crassifolia*	CMW38885	MN644475	MN644475	MN647808
*Grosmannia curvispora*	CBS123914	MN644473	MN644473	MN647806
*Grosmannia penicillata*	CMW2642	MN644478	MN644478	–
*Harringtonia aguacate*	Raff.sp.272	MT633065	MT629748	–
*Harringtonia ambrosioides*	**18055**	**ON145696**	**ON142055**	**ON142055**
Harringtonia ambrosioides *	**FLAS-F-70270**	**–**	**ON142057**	**ON142057**
*Harringtonia ambrosioides*	**18056**	**ON145697**	**ON142056**	**ON142056**
Harringtonia arthroconidialis *	**FLAS-F-70272**	**ON145695**	**ON142054**	**ON142054**
*Harringtonia brunnea*	CBS378.68	–	EU984284	EU977460
Harringtonia chlamidospora *	**FLAS-F-70271**	**–**	**ON142062**	**ON142062**
*Harringtonia chlamidospora*	**18110**	**–**	**ON142061**	**ON142061**
*Harringtonia chlamidospora*	**FLAS-F-70273**	**–**	**ON142060**	**ON142060**
*Harringtonia lauricola*	C2339	–	EU123077	–
*Harringtonia lauricola*	PL159	–	KJ909303	KJ909302
*Harringtonia lauricola*	Raff.sp.570	MT633071	MT629759	MT644093
*Harringtonia sporodochialis*	**18073**	**ON145698**	**ON142058**	**ON142058**
Harringtonia sporodochialis *	**FLAS-F-70269**	**–**	**ON142059**	**ON142059**
*Leptographium aenigmaticum*	CMW2199	AY553389	–	AY534937
*Leptographium alacris*	CMW2844	JN135313	JN135313	JN135329
*Leptographium clavigerum*	ATCC18086	AY544613	–	–
*Leptographium francke-grosmanniae*	CMW445	MN516715	MN516715	–
*Leptographium koreanum*	MCC206	AB222065	AB222065	AB222063
*Leptographium laricis*	CMW1980	DQ062074	DQ062074	DQ062008
*Leptographium longiclavatum*	SL Kw1436	AY816686	–	AY288934
*Leptographium lundbergii*	UAMH9584	AY544603	–	AY263184
*Leptographium lundbergii*	CMW2190	DQ062066	DQ062066.1	DQ062000
*Leptographium lundbergii*	CBS 352.29	MH855083	MH866542	–
*Leptographium neomexicanum*	CBS168.93	NR_160191	MH874049	–
*Leptographium olivaceum*	CBS138.51	NR_155106	MH868302	–
*Leptographium profanum*	CMW10552	DQ354944	DQ354944	DQ354936
*Leptographium serpens*	CMW304	JN135314	JN135314	JN135334
*Leptographium sibiricum*	CMW4481	KM491424	KM491424	KM491378
*Leptographium terebrantis*	UAMH9722	AY544606	–	AY263192
*Leptographium wageneri*	AF343708	AF343708	–	–
*Leptographium wingfieldii*	CMW2096	AY553398	AY553398	AY707191
*Ophiostoma pilliferum*	AU55-4	AF221073	AF221624	–
*Raffaelea albimanens*	CBS271.70	MT633066	EU984296	MT644111
*Raffaelea ambrosiae*	CMW25533	MT633068	MT629752	MT644095
*Raffaelea ambrosiae*	CBS185.64	MT633067	MT629751	MT644094
*Raffaelea arxii*	CBS273.70	MH859604	MT629753	–
*Raffaelea borbonica*	CMW51548	MT633054	MT629736	MT644100
*Raffaelea canadensis*	CBS168.66	GQ225699	–	EU977473
*Raffaelea canadensis*	CMW25536	–	MT629755	–
*Raffaelea cyclorhipidia*	CMW44790	MT633069	MT629757	–
*Raffaelea ellipticospora*	C2709	–	HQ688664	–
*Raffaelea ellipticospora*	CMW38056	MT633070	MT629758	–
*Raffaelea ellipticospora*	C2345	–	–	KJ909298
*Raffaelea fusca*	C2394	–	EU177449	KJ909301
*Raffaelea gnathotrichi*	C2219	–	EU177460	–
*Raffaelea santoroi*	CMW25539	MT633075	MT629765	–
*Raffaelea santoroi*	CBS399.67	–	EU984302	EU977476
*Raffaelea seticollis*	CMW1031	MT633076	MT629766	–
*Raffaelea subalba*	C2401	–	EU177443	KJ909305
*Raffaelea subfusca*	C2335	–	EU177450	KJ909307
*Raffaelea sulcati*	CBS806.70	–	–	EU977477
*Raffaelea sulcati*	C2234	–	EU177462	–
*Raffaelea tritirachium*	CBS726.69	–	EU984303	EU977478
*Raffaelea xyleborina*	Hulcr6099	–	–	KX267124
*Raffaelea xyleborina*	CMW45859	MT633078	MT629769	–
*Sporothrix eucalyptigena*	TYPE	NR137979	NG058162	MG431426

## Data Availability

Genetic data were deposited in GenBank and can be accessed using the numbers provided in Table 1. Any further request relating to the data please contact J.P.M.A.
